# Incidence and Risk Factors of Clinical Deterioration during Inter-Facility Transfer of Critically Ill Patients; a Cohort Study

**Published:** 2020-07-04

**Authors:** Kannapatch Srithong, Siriorn Sindhu, Napaporn Wanitkun, Chukiat Viwatwongkasem

**Affiliations:** 1 DNS candidate, Faculty of Graduate Studies,Mahidol University, Thailand.; 2 RN, PhD, Associate Professor, Department of Surgical nursing, Faculty of Nursing,Mahidol University, Thailand.; 3 RN, PhD, Assistant Professor, Department of Surgical nursing, Faculty of Nursing,Mahidol University, Thailand.; 4 PhD, Associate Professor, Biostatistics Department, Faculty of Public Health,Mahidol University, Thailand.

**Keywords:** Patient transfer, critical illness, clinical deterioration, Thailand

## Abstract

**Introduction:**

Critically ill and injured patients are at a higher risk of developing clinical deterioration during inter-facility transfers. This study aimed to determine the incidence rate and risk factors of clinical deterioration among critically ill patients during inter-facility transfers in Thailand.

**Methods:**

The present cohort study was conducted in 22 referring hospitals and 7 receiving hospitals under the supervision of Ministry of Public Health, Thailand, between March 15 and December 31, 2018. The subjects were comprised of 839 critically ill patients aged 18 and over, 63 coordinator nurses in referral centers, and 312 referral team leaders. Data collected included pre-transfer risk score, clinical data of patient during transfer, characteristics of referral team leader, ambulance type, preparation time, time to definitive care, transfer distance, and National Early Warning Score (NEWS) (clinical deterioration). Multilevel mixed-effects regression analysis was performed.

**Results:**

The incidence rate of clinical deterioration was 28.69%. The most common types of clinical deterioration were hemodynamic instability, respiratory instability, and neurological alteration. Time between 31-45 minutes was significantly associated with clinical deterioration (β 0.133, *P* value 0.027). The following illnesses were associated with higher probability of clinical deterioration: body region injuries/head injury/burn/ingested poison (β 0.670, *P* value 0.030), respiratory distress/convulsion (β 0.919, *P* value 0.001), shock/ arrhythmias/chest pain/hemorrhage (β 1.134, *P* value <0.001), comatose/alteration of consciousness/syncope (β 1.343, *P* value <0.001), and post-cardiac arrest (β 2.251, *P* value <0.001). Patients with unstable conditions (β 1.689, *P* value 0.001) and pre-transfer risk score of 8 or higher (β 0.625, *P* value 0.001) had a higher rate of deterioration. Transfer by non- emergency room (ER) nurses (β 0.495, *P* value 0.008) and transportation in a mobile intensive care unit (ICU) were associated with a higher rate of deterioration (β 0.848, *P* value 0.001).

**Conclusion:**

The incidence of clinical deterioration during inter-facility transfer in Thailand was high. Illnesses involving circulatory, respiratory, and neurological systems, clinical instability, high pre-transfer risk score, transport time of 31-45 minutes, transportation by non-ER nurse, and mobile ICU were associated with a higher rate of clinical deterioration.

## Introduction

Clinical deterioration, including cardiac arrest, can occur during inter-facility transfer of critically ill patients. Recent evidence on the incidence rate of clinical deterioration during inter-facility transfer is quite limited. Two studies conducted in the Netherlands reported incidence rates between 15.2 – 34.0% (1, 2). Two studies in Canada and Saudi Arabia reported the incidence rates of 6.5% and 13.6%, respectively (3, 4). A number of risk factors for inter-facility clinical deterioration have been examined in international literature. These factors can be classified into patient-related factors and service-related factors (1-5). 

Clinical deteriorations have been found to be associated with age, sex, clinical conditions, and level of patient acuity (4, 5). Increasing age, being male, sustaining illnesses involving circulatory, respiratory, and neurological systems, and having high acuity increase the risk of clinical deteriorations. The service factors included pre-transfer risk score, during-transfer care, characteristics of referral team leader, ambulance type, preparation time, time to definitive care, and transfer distance (1-4, 6). 

Higher pre-transfer risk score predicts higher probability of clinical deterioration (3, 6). Patients receiving appropriate management until achieving clinical stabilization before transfer are less likely to experience clinical deterioration (4, 5, 7). During-transfer care includes assessment and monitoring of the patient as well as interventions given to the patient. In the Netherlands, Ligtenberg et al. (2006) reckoned that the lack of during-transfer care protocols contributed to high incidence of clinical deterioration (1). A subsequent Dutch study, which incorporated a during-transfer protocol, reported a much lower incidence rate for clinical deterioration (2). During the transfer, patients managed by doctors, nurses, and paramedics who underwent critical care transport training were less likely to experience clinical deterioration (2, 3). Patients being transported in an ambulance fully equipped with critical care technologies (i.e., mobile intensive care unit (ICU)) were less likely to experience clinical deterioration (2, 7). Increasing preparation time, time to definitive care, and transfer distance increase clinical deterioration incidence (4). Recent studies have not found an association between clinical deterioration and post-transfer outcomes such as ICU admission and death (3, 8). This may be due to limitations related to sampling and outcome measurement. Nonetheless, in hospitalized patients, clinical deterioration is often associated with more serious health outcomes such as ICU admission and death. Clinical deterioration is still an issue of great concern. Approximately 52-91% of clinical deterioration incidents are preventable. Clinical deterioration can be prevented via early detection (9). Early detection of clinical deterioration leads to timely management of deteriorating changes and therefore, prevents progression to fatal events such as cardiopulmonary arrest.

 In developed countries, inter-facility transfer of critically ill patients is carried out either by well-equipped ambulance centers or tertiary care hospitals (2, 7, 10-12). Staff responsible for transporting the patients in these countries must undergo critical care transport training to ensure suitable knowledge and skills in managing patients’ conditions and clinical deterioration (2, 3). In Thailand, transfers of critically ill patients is carried out by resource-limited community hospitals. These resource-deprived community hospitals rely on emergency room (ER) nurses and non-ER nurses with varying qualifications and experiences when transporting the patients. The majority of these nurses do not have formal training in inter-facility transfer. Lack of knowledge and skills among the referring nurses can compromise the ability of nurses in detecting early warning signs, managing deterioration, and preventing and managing these conditions during the transfer. Variations in patient transfer structures, practices and outcomes can be expected due to varying material and human resources as well as the lack of consensus practice guidelines among these hospitals. 

Since clinical deterioration during the transfer can be harmful, data about its incidence and risk factors are important for health policymakers and practitioners to understand the situation and note gaps in existing inter-facility transfer services. Such knowledge is needed for evidence-based decision making to improve regulations, structural support, and human resource development to improve early detection, prevention, and management of patient deterioration during the transfer. This cohort study aimed to determine the incidence and risk factors of inter-facility transfer clinical deterioration.

## Methods

***2.1 Study design and setting***

This cohort study took place in Thailand, between March 15 and December 31, 2018. Stratified random sampling was used. Seven health service networks were randomly selected from 12 networks (excluding Bangkok Metropolitan Administration). Seven provinces were then selected from these networks. Based on the proportion, two to six community hospitals within these provinces were then randomly recruited into the study as referring hospitals. In case there were more than one receiving hospitals in the province, the most advanced hospital was selected as receiving hospital. The study involved 22 referring hospitals and 7 receiving hospitals under the supervision of Ministry of Public Health across Thailand. This study concerned only inter-facility transfers that took place within the province (i.e., intra-province transfers). Transfers between provinces were not included in this study. The study protocol was approved by the Institutional Review Board, Faculty of Nursing, Mahidol University (No: IRB-NS2017/423.2512). To comply with local human research ethics requirements, additional ethics approvals were also sought from seven receiving hospitals before the commencement of the study. 

***2.2 Participants***

Critically ill patients, referring nurses, and coordinator nurses comprised the study samples. The patient sample size calculation was based on power of analysis, level of significance, effect size, intraclass correlation (ICC) coefficient, and possible sample size of the participants (13). First, we determined the effect size (d) based on data from the previous study on inter-facility transfer (14) with power of analysis and level of significance of 0.80 and 0.05, respectively. Second, we determined the sample size of the referring hospitals based on the desired intra-class correlation (ICC) of 0.30 and the expected sample size of 30 patients per hospital. The calculation resulted in the referring hospital sample size of 22. The patient sample size was, therefore, 660 (i.e., 30 x 22). To deal with potential loss of data, we increased the sample size by 30% or 198 patients. After rounding up, the final sample size was 880. 

***2.3 Inclusion and exclusion criteria***

***Patients***

To be included in the study, the patients had to be at least 18 years of age, treated in the emergency room at referring hospitals, and critically ill as per patient acuity classification. Patients diagnosed with psychiatric and obstetric emergencies were excluded. 

***Referring and coordinators nurses***

As per the national clinical practice guideline, at least two health practitioners are assigned to the transfer of critically ill patients. Only the nurses who were designated as team leader of the inter-facility transfer team were included in the study regardless of their experience and qualifications. Coordinator nurses who were on duty at the receiving hospitals during the transfer were included. 

**2.4 Variables**

***Independent variables measurement***

Independent variables included patient sex and age, level of patient acuity, clinical signs and symptoms, and pre-transfer risk score, referring nurse’s qualification, referring nurse’s experience, preparation time, type of ambulance, during-transfer reassessment, transfer distance, transfer time, and time to definitive treatment. Pre-transfer risk score was assessed using Risk Score of Interhospital Transport of Critically Ill Patients (RSTP) (6). An RSTP score of 8 or higher was considered as an indication of high risk for clinical deterioration. Inter-rater reliability of RSTP among the researcher and 14 research assistants was 0.83 based on Kappa coefficient (15). Nurse’s qualifications were described as emergency nurse practitioner (ENP), ER nurse, and non-ER nurse. Nurse’s inter-facility transfer experience was measured as number of years involved in interfacility transfer (IFT). Preparation time was the duration between the time when the patient first presented to ER to the time when referral decision was made. Time to definitive treatment was the duration between the time when the referral decision was made to the time when the patient received treatment at the receiving hospital. These times were measured in minutes. Transfer distance was the distance between referring hospital and receiving hospital and was measured in kilometers. Content validity of other instruments was obtained through a panel of experts. These data were collected from inter-facility transfer records from referring and receiving hospitals as well as on the ambulance. 

***Outcome measurement***

The studied outcome was clinical deterioration. The following clinical conditions occurring during the transfer was defined as incidence of clinical deterioration: systolic blood pressure (SBP) < 90 mmHg or > 160 mmHg, SpO_2_ < 92% for intubated patients or < 90% or baseline reduction ≥ 5% in adults, respiratory rate ≥ 26 bpm or ≤ 10 bpm, heart rate ≥ 140 bpm or ≤ 40 bpm, increased chest pain, arrhythmias, cardiac arrest, a drop in Glasgow Coma Score ≥ 2, and convulsion. Patients who experienced any of the above-mentioned conditions at any given time during the transfer were regarded as having clinical deterioration. The National Early Warning Score (NEWS) was used to assess changes in clinical deterioration score. Internationally, clinical deterioration can be detected using different tools such as Rapid Emergency Medicine Score (REM), Early Warning Score (EWS), Risk Score for Transfer Patient (RSTP), Modified Early Warning Score (MEWS), Therapeutic Intervention Scoring System (TISS-28), and National Early Warning Score (NEWS) (6, 16-19). Among these instruments, NEWS is the most effective in predicting serious clinical deterioration and was found to be sensitive to cardiopulmonary arrest (19). This study, therefore, used NEWS to assess patient deterioration. The score was derived from the assessment of 7 physiological parameters as follows: respiratory rate, oxygen saturation, supplemental oxygen, temperature, systolic blood pressure, heart rate, and level of consciousness (19, 20). NEWS parameters were recorded by referring nurse in the patient transfer form at six different time intervals throughout the transfer, namely, 1-15 minutes, 16-30 minutes, 31-45 minutes, 46-60 minutes, 61-75 minutes, and 76-90 minutes. The researcher and research assistants then retrieved the data from the record form.

***2.5 Statistical analysis***

Multilevel mixed-effects linear regression analysis was done using STATA statistical software version 16.0. Findings were presented as a model of factors predicting clinical deterioration during inter-facility transfer. *P* values less than 0.05 were considered statistically significant.

## Results

***3.1 Baseline characteristics of inter-facility transfer system***

839 critically ill patients comprised the final patient sample in this study. The majority were male (60.67%). The mean age was 59.57 ± 18.00 (18 - 99) years. The majority suffered from critical illnesses involving circulatory and respiratory systems (57.45%) and were classified as stable with high risk of deterioration (84.27%). 60.67% of the patients were considered as having a high risk for clinical deterioration. The majority of referring team leaders were ER nurses (55.45%). The mean inter-facility transfer experiences were 9.51 ± 7.54 (<1-36) years. The mean preparation time and time to definitive treatment were 72.10 ± 28.35 (12-120) and 52.38 ± 21.97 (16-170) minutes, respectively. Transfer distance was 52.97 ± 28.31 (8.9-110.0) kilometers.

***3.2 Clinical deterioration***

The incidence of clinical deterioration during inter-facility transfer was 28.37% (n = 238/839). Unstable patients had a higher rate of clinical deterioration (63.27%; n = 62) compared to stable patients with a high risk of deterioration (24.33%; n = 172) and stable patients with a medium risk of deterioration (11.76%; n = 4). Overall, the average NEWS of critically ill patient sample during each timeframe was between 4.57 to 4.90. The NEWS of individual patients ranged from 0 to 20. The highest average score was 4.90 ± 2.77, which was measured during 31-45 minutes. Considering clinical stability, stable patients with high risk of deterioration had an average NEWS between 4.69 and 4.92 with the highest average score of 4.92 ± 2.80 at 76-90 minutes. High average NEWS (between 6.35 and 7.12) was observed among unstable patients with the highest average score of 7.12 ± 3.20 at 46-60 minutes.

**3.3 Predictors of clinical deterioration during transfer**

The following variables, which were found to have significant association (*P* value < 0.25) with clinical deterioration in a univariate analysis, were included in subsequent multilevel mixed-effects linear regression analysis: patient sex, clinical signs and symptoms of illnesses, patient acuity level, pre-transfer risk score, transfer team leader’s qualification, transfer team leader’s experience, during transfer reassessment, ambulance types, preparation time, time to definitive treatment, and transfer distance. In this study, individual patients with critical illness had an average NEWS of 2.403 throughout the transfer. Clinical signs and symptoms involving circulatory, respiratory, and neurological systems, unstable conditions, pre-transfer high risk score, transfer by non-ER nurses, transport by Mobile ICU, and 31-45 minutes transfer timeframe were found to be associated with a significant increase in NEWS (Table 3).

**Table 1:** Characteristics of inter-facility transfers of critically ill patients

**Variables**	**Values**
***Patient demographics***	
**Sex**	
Male	509 (60.67)
Female	330 (39.33)
**Age (year)**	
Mean ± SD (range)	59.57±18.00 (18-99)
**Clinical signs and symptoms of illness **	
(a) Hemiplegia/Paraplegia/Severe pain/High fever	110 (13.11)
(b) Body region injuries/Head injury/Burn/Ingested poison	113 (13.47)
(c) Respiratory distress/Convulsion	280 (33.37)
(d) Shock/Arrhythmia/Chest pain/Hemorrhage	202 (24.08)
(e) Comatose/Alteration of consciousness/Syncope	95 (11.32)
(f) Post cardiac arrest	39 (4.65)
**Patient acuity level**	
Stable with medium risk of deterioration	34 (4.05)
Stable with high risk of deterioration	707 (84.27)
Unstable with clinical deterioration	98 (11.68)
**Pre-transfer risk score **	
Low risk (≤7 scores)	330 (39.33)
High risk (≥8 scores)	509 (60.67)
Mean ± SD (range)	8.00±2.61 (0-20)
***Inter-facility service demographics***	
**Transfer team leader’ qualification** (n=312)	
Non-ER nurse	98 (31.41)
ER nurse	173 (55.45)
ENPs or ENs	41 (13.14)
**Transfer team leader’s experience (year)**	
Mean ± SD (range)	9.51±7.54 (1-36)
**Ambulance types **	
EMS ambulance	622 (74.14)
Mobile ICU ambulance	217 (25.86)
**D** **uring transfer reassessment**	
More than 15 minutes	306 (36.47)
Every 5-15 minutes	533 (63.53)
**Preparation time** **(minutes)**	
Mean ± SD (range)	72.10±28.35 (12-120)
**Time to definitive** **treatment (minute) **	
Mean ± SD (range)	52.38±21.97 (16-170)
**Transfer distance** **(kilometer)**	
Mean ± SD (range)	59.97±28.31 (8.9-110.0)

Data are presented as mean ± standard deviation (SD) or frequency (%). ER: emergency room, ENP: emergency nurse practitioner, EN: emergency nurse, EMS: emergency medical service, ICU: intensive care unit.

**Table 2:** National Early Warning Score (NEWS) classified by time of measurement and patient acuity level

**NEWS** **(** **0** **-** **20** **)**		**Time of measurement**					
	**Time 0**	**Time 1**	**Time 2**	**Time 3**	**Time 4**	**Time 5**	**Time 6**
	**before transfer**	**1** **-** **15 minutes**	**16** **-** **30** **minutes**	**31** **-** **45** **minutes**	**46** **-** **60** **minutes**	**61** **-** **75 minutes**	**76** **-** **90** **minutes**
**Total critically ill patients **	(n=839)	(n=839)	(n=776)	(n=657)	(n=497)	(n=258)	(n=80)
Mean ± SD	4.70±2.66	4.74±2.75	4.73±2.76	4.90±2.77	4.84±2.85	4.57±2.70	4.86±3.02
Range	(0 to 14)	(0 to 18)	(0 to 18)	(0 to 20)	(0 to 20)	(0 to 15)	(0 to 13)
**Stable with medium risk of deterioration **	(n=34)	(n=34)	(n=32)	(n=25)	(n=25)	(n=16)	(n=4)
Mean ± SD	0.00±0.00	0.21±0.64	0.31±1.00	0.32±1.14	0.24±1.01	0.00±0.00	0.00±0.00
Range	(0)	(0 to 3)	(0 to 4)	(0 to5)	(0 to 5)	(0)	(0)
**Stable with high risk of deterioration **	(n=707)	(n=707)	(n=662)	(n=564)	(n=422)	(n=222)	(n=66)
Mean ± SD	4.64±2.43	4.69±2.05	4.74±2.59	4.89±2.56	4.84±2.53	4.74±2.44	4.92±2.80
Range	(3 to 13)	(2 to 13)	(3 to 17)	(3 to 17)	(3 to 17)	(2 to 15)	(3 to 13)
**Unstable with clinical deterioration **	(n=98)	(n=98)	(n=82)	(n=68)	(n=50)	(n=20)	(n=10)
Mean ± SD	6.67±2.56	6.72±2.90	6.35±2.74	6.68±2.91	7.12±3.29	6.40±2.87	6.40±3.20
Range	(3 to 14)	(3 to 18)	(3 to 18)	(4 to 20)	(4 to 20)	(3 to 11)	(3 to 13)

SD: standard deviation.

**Table 3:** Multilevel mixed-effects analysis to predict clinical deterioration

**Variables**	**Multivariable analysis**					
	$\overset{\text{\textasciicircum{}}}{\beta }$	**95%CI**			***P*** ** value**	
**Fixed effect**						
Intercept (*β*_0_)	2.403	(1.580	3.226)		<0.001	
**NEWS Score during-transfer time**						
Time 0 (Before transfer)	ref.					
Time 1 (1-15 minutes)	0.037	(-0.071	0.145)		0.504	
Time 2 (16-30 minutes)	0.104	(-0.007	0.216)		0.067	
Time 3 (31-45 minutes)	0.133	(0.015	0.251)		0.027*	
Time 4 (46-60 minutes)	0.106	(-0.025	0.236)		0.112	
Time 5 (61-75 minutes)	0.009	(-0.158	0.176)		0.918	
Time 6 (76-90 minutes)	0.242	(-0.036	0.520)		0.087	
**Clinical signs and symptoms of illness**							
Hemiplegia/Paraplegia/Severe pain/High fever	ref.			
Body region injuries/Head injury/Burn/Ingested poison	0.670	(0.065	1.274)		0.030*		
Respiratory distress/Convulsion	0.919	(0.371	1.467)		0.001*		
Shock/Arrhythmias/Chest pain/Hemorrhage	1.134	(0.569	1.698)		<0.001*		
Comatose/Alteration of consciousness/Syncope	1.343	(0.661	2.026)		<0.001*		
Cardiac arrest	2.251	(1.175	3.328)		<0.001*		
**Patient Acuity Level**							
Stable with medium risk of deterioration	ref.						
Stable with high risk of deterioration	0.651	(-0.172		1.475)	0.121
Unstable with clinical deterioration	1.689	(0.663		2.715)	0.001*
**Pre-transfer risk score **					
RSTP ≤7 scores	ref.		
RSTP ≥ 8 scores	0.625	(0.269		0.981)	0.001*
**Referral team leader**					
ER nurses	ref.				
Non-ER nurses	0.495	(0.132		0.857)	0.008*
ENPs or ENs	0.064	(-0.519		0.647)	0.829
**Ambulance** ** type**					
Standard ambulance	ref.				
Mobile ICU	0.848	(0.349		1.347)	0.001*

* Significant *P* value. NEWS: National Early Warning Score; RSTP: Risk Score for Transport Patients, ER: emergency room; ENP: emergency nurse practitioner, EN: emergency nurse, ICU: intensive care unit; ref.: reference.

## Discussion

This study examined clinical deterioration of critically ill patients during inter-facility transfer. The inter-facility clinical deterioration rate was 28.37% (n = 238). Symptoms of clinical deterioration could be classified into hemodynamic instability, respiratory instability, and neurological alteration. The classification resembled previous studies in Canada, Saudi Arabia, and Australia (3, 4, 10). 

The major results described patient, service, and time-related factors associated with patient deterioration as measured by NEWS. Illnesses involving circulatory, respiratory, and neurological systems significantly increased clinical deterioration incidences when compared with motor deficit/severe pain/high fever. Unstable conditions significantly increased NEWS by 1.689 compared with stable conditions with a medium risk of deterioration. High pre-transfer risk score (≥ 8) significantly increased the NEWS by 0.625 compared with low pre-transfer risk score (≤ 7). A significant increase of NEWS by 0.133 was found 31-45 minutes after transfer when compared with NEWS before transfer. Transfer by non-ER nurses increased the NEWS by 0.495 when compared to the transfer by ER nurses. Transfer by mobile ICU increased NEWS by 0.848 when compared with transfers by standard ambulance.

In our study, it is clear that patients having circulatory, respiratory, and neurological illnesses, unstable clinical conditions and pre-transfer risk score were more likely to experience clinical deterioration during the transfer. In a previous study by Alabdali et al. (2017), cardiac patients were less likely to develop clinical deterioration (adjusted OR: 0.117, 95% CI: 0.02 to 0.52 and *p* value <0.01) (3). In another study, cardiac patients (crude OR: 0.6, 95% CI: 0.4 to 0.8), neurologic patients (crude OR: 0.4, 95% CI: 0.3 to 0.6), and trauma patients (crude OR: 0.5, 95% CI: 0.3 to 0.8) were less likely to develop clinical deterioration (4). Our findings appear to contradict with previous studies. It is, however, of note that the way we categorized patients’ illness in this study differed from that of the previous two studies. Our study used clinical signs and symptoms as a basis for illness categorization whereas the previous studies relied on medical diagnoses. Clinical signs and symptoms and medical diagnoses are not always the same. Patients with cardiac diagnosis, for example, do not always present with cardiac signs and symptoms. This also applies to patients with neurologic and trauma diagnoses. We believe that clinical signs and symptoms better reflect patient conditions and needs, since they change during critical illness unlike medical diagnosis. Clinical signs and symptoms offered a better basis for illness categorization as they were more sensitive to clinical deterioration as evident in this study; whereas, medical diagnoses were not. In addition, clinical signs and symptoms help guide appropriate interventions related to physiological changes, which are common in critically ill patients. 

Patient acuity and pre-transfer risk score could predict patient deterioration during the transfer. This is not surprising as these variables were derived from data concerning clinical deterioration, for example, low blood pressure, low heart rate, decreased level of consciousness, increased respiratory rate, oxygen desaturation, and cardiac arrest. Our findings were consistent with previous studies in Canada, Saudi Arabia, and Hong Kong (3-5). Lee et al. (2010) reported that physiological instability before the transfer was a significant risk factor of clinical deterioration (*P *value 0.004). Singh et al. (2014) reported that patients with baseline hemodynamic instability were more likely to develop clinical deterioration during the transfer (crude OR: 3.9, 95% CI: 3.1 to 4.9). Alabdali et al. (2017) reported that patients with pre-transfer risk score of 6 or higher were 1.3 times more likely to experience clinical deterioration. Our study has confirmed that patient acuity and pre-transfer risk score were predictive of clinical deterioration, and thus can be used as tools to anticipate changes in patient conditions and plan for proper prevention and management during the transfer.

Only one study identified the effect of time on clinical deterioration. Clinical deterioration increased 1.15 times for each additional 10 minutes of transport time (4). Longer ride leads to accumulative effects of physiological changes associated with vibrations and inertial force during ambulance moving and these effects can contribute to the occurrence of deterioration (11). Acceleration, deceleration, and vibrations in a moving car are associated with hemodynamic changes. These changes include low blood pressure, interference with blood flow to the brain, and increased heart rate and respiratory rate (12). 

Our study also found the effect of time on clinical deterioration. However, a significant increase of NEWS was found only 31-45 minutes after departure when compared with departure time. Upon close examination, we found that the increase in NEWS 31-45 minutes after departure was due to the occurrence of serious clinical deterioration due to cardiac arrest, hypotension, oxygen desaturation, and alteration of consciousness in this period. Why these serious events occurred more frequently during this period warrants explanation. Due to limited knowledge about this occurring, we attempt to hypothesize this for future research. First, the adverse effects of certain medical treatments received at the referring hospital may fall within this period. Such treatments are, for example, thrombolytic agent and bronchodilator. Peak of action of bronchodilator is between 30-60 minutes. The adverse reactions that can present during this time frame include palpitation, hypertension, tachycardia, chest pain, dyspnea, and paradoxical bronchospasm. Streptokinase, is known to induce arrhythmias and hypotension, which normally occur during the first 15-30 minutes after infusion. In Thailand, streptokinase is infused for patients with myocardial infarction at the referring community hospital before departure. Currently there are two common practices regarding streptokinase infusion. First, the referring hospital completes the infusion before commencing the transfer. Second, the referring hospital commences the transfer immediately after starting the infusion. The latter practice might lead to more frequent occurrence of clinical deterioration due to unmanaged adverse drug reactions during the early stages of transfer. 

Another explanation concerns the sustainability of therapeutic effects of medical treatments received at the referring hospital. For example, optimal blood pressure in patients with septic shock and trauma can be achieved through fluid resuscitation in ER. However, this resuscitative effect is best seen during the first 30 minutes and only lasts for 60 minutes. This may reintroduce clinical instability (i.e., hypotension) during transfer, especially when there is no or inadequate intervention to maintain optimal hemodynamics. Our data showed that a number of critically ill patients developed new and recurrent clinical deterioration throughout the transfer.

The patients transferred by non-ER nurses were more likely to experience clinical deterioration compared to those transferred by ER nurses and ENPs. As per the quality mandate in Thailand, all ER nurses must undergo compulsory advanced life support annually. In-house trainings related to pre-hospital care, inter-facility transfer and mass casualty management were also very common among these nurses. In addition, ER nurses were involved in inter-facility transfers on a much more regular basis than non-ER nurses. The ENPs, on the other hand, completed a four-month intensive training program plus continuing staff development activities undertaken by ER nurses. This set of knowledge and skills acquired through trainings and experiences made the ER nurses and ENPs more efficient in managing critically ill patients during the transfer, compared to non-ER nurses who rarely underwent these trainings and infrequently attended to critically ill patients. Our finding was consistent with those described in previous studies. Patients transferred by paramedics with training in critical care transport experienced clinical deterioration significantly less than those transferred by paramedics without critical care transport (3). This finding was indicative of the role of knowledge and skills of transferring personnel in prevention and management of clinical deterioration.

This study was the first to examine the role of ambulance type on clinical deterioration. Our original hypothesis was that patients transferred by standard ambulance would be at higher risk of experiencing clinical deterioration than those transferred by a mobile ICU. To our surprise, the result was the opposite. Our study revealed that transfer by mobile ICU ambulance increased NEWS by 0.848 when compared to transfer by standard ambulance. We reckon that the increase in clinical deterioration rate in mobile ICU ambulance reflected the detectability rather than the actual occurrence of clinical deterioration. In a standard ambulance with no continuous monitoring, it was likely that fewer signs of clinical deterioration were detected and recorded than they actually occurred. Continuous monitoring in mobile ICU ambulance facilitated rapid detection and documentation of abnormal changes in physiologic parameters such as heart rate, respiration, blood pressure and oxygen saturation. 

Sex and age of the patients, preparation or resuscitation time, frequency of patient reassessment during transfer, inter-facility transfer experience of nurses, transfer distance, and time to definitive treatment were not found to be associated with clinical deterioration. We expected to find the association between age and clinical deterioration as in a previous study (4), however, our result was different. Sex was not also associated with clinical deterioration. These variables might have served as confounders in this study. Referral experience of nurses, which was measured as number of years involved in inter-facility patient transfer was not associated with clinical deterioration. Preparation time was not associated with the increase in NEWS despite our previous understanding that preparation time could suggest patient severity, which would result in higher probability of subsequent deterioration. In the Thai contexts, referring hospitals are expected to stabilize the patient before they initiate the transfer. This preparation period may be short or long, depending on whether the patients achieve stabilization goals. Preparation time was, therefore, not an important factor in determining patient safety (i.e., clinical deterioration) but achievement of stable conditions before departure was. For us, the number of years in inter-facility transfer did not equate expertise in inter-facility transfer, which was found to affect clinical deterioration incidence. This is especially true for non-ER nurses who did not have enough training and were put in charge of patient transfer once in a while, even though they had been involved in inter-facility transfer for a number of years. It is, therefore, important that we consider expertise but not the number of practice years when choosing nurses for patient transfer. 

The Canadian study revealed the effect of transfer distance on clinical deterioration (4). In our study, the distance was not associated with clinical deterioration. The longest distance in this study, which concerned only within-provincial transport, was 110 KMs; whereas, the shortest was 8.9 KMs. From the result, we could only say that the increase in distance did not increase clinical deterioration incidence, when the distance was between 8.9-110 KMs. It is of practical note to point out that patient transport involving distances longer than 110 KMs, as in inter-provincial transport, may not be as safe. The longer distance might produce different results (i.e., increase in clinical deterioration) and system redesign may be needed to ensure safety during transport in such situations. This is, however, subject to further investigation.

The increase in time to definitive treatment did not increase NEWS. All the patients in this study reached the receiving hospitals within 170 minutes since the referring hospital contacted the receiving hospital for referral. Again, we could only say that this timeframe did not result in a higher rate of clinical deterioration. Further study is required to determine whether it is still as safe beyond this timeframe, or not. 

Clinical deterioration during inter-facility transfer of critically ill patients can be better prevented and managed by pre-departure assessment of clinical deterioration risks using associated clinical parameters such as patient acuity and signs and symptoms, adequate stabilization of the patients, designation of ENP or ER nurses as transfer leader, close monitoring of deteriorating signs through mobile ICU, and continuing management during transport. 

## Limitation:

Limitations concerning the completeness of clinical data recorded during transfer might have been present. Despite our briefing on documentation requirements, variations in nurses' knowledge and skills could have impacted the quality of documentation and thus, quality of the data collected.

## Conclusion

The incidence of clinical deterioration during inter-facility transfer of critically ill patients between community hospitals and provincial hospitals in Thailand was 28.7%. Patients with signs and symptoms involving circulatory, respiratory, and neurologic systems, unstable clinical conditions, and high pre-transfer risk scores were at risk of experiencing clinical deterioration. Achievement of stabilization before departure, regardless of preparation time, was a safe practice. Patients transferred by ENPs did not experience an increase in clinical deterioration rate; whereas, those managed by non-ER nurses did. Mobile ICU detected more deterioration incidents than standard ambulances. An increase in clinical deterioration rate could be expected 31-45 minutes after departure from the referring hospital. A timeframe between 16-170 minutes and transfer distance between 8.9-110 KMs did not cause any differences in clinical deterioration rate. Within the context of our study, these time and distance limits were, therefore, considered reasonably safe for patient transport.
